# Burstiness and tie activation strategies in time-varying social networks

**DOI:** 10.1038/srep46225

**Published:** 2017-04-13

**Authors:** Enrico Ubaldi, Alessandro Vezzani, Márton Karsai, Nicola Perra, Raffaella Burioni

**Affiliations:** 1 ISI Foundation, 10126 Torino, Italy; 2 Dipartimento di Fisica e Scienza della Terra, Università di Parma, Parco Area delle Scienze 7/A, 43124 Parma, Italy; 3 INFN, Gruppo Collegato di Parma, Parco Area delle Scienze 7/A, 43124 Parma, Italy; 4 CNR, IMEM, Parco Area delle Scienze 37/A, 43124 Parma, Italy; 5 Univ Lyon, ENS de Lyon, INRIA, CNRS, UMR 5668, IXXI, 69364 Lyon, France; 6 Centre for Business Network Analysis, University of Greenwich, Park Row, London SE10 9LS, United Kingdom

## Abstract

The recent developments in the field of social networks shifted the focus from static to dynamical representations, calling for new methods for their analysis and modelling. Observations in real social systems identified two main mechanisms that play a primary role in networks’ evolution and influence ongoing spreading processes: the strategies individuals adopt when selecting between new or old social ties, and the bursty nature of the social activity setting the pace of these choices. We introduce a time-varying network model accounting both for ties selection and burstiness and we analytically study its phase diagram. The interplay of the two effects is non trivial and, interestingly, the effects of burstiness might be suppressed in regimes where individuals exhibit a strong preference towards previously activated ties. The results are tested against numerical simulations and compared with two empirical datasets with very good agreement. Consequently, the framework provides a principled method to classify the temporal features of real networks, and thus yields new insights to elucidate the effects of social dynamics on spreading processes.

The recent availability of longitudinal and time-resolved datasets capturing social behaviour has induced a paradigm shift in the way we study, describe, and model the interactions between individuals. It moved the focus from static, time-aggregated, representations to time-varying, dynamical, characterisations of social networks^1^,^2^,^3^,^4^. Thinking in terms of time-varying systems allows to overcome the limitations arising from the depiction of social ties as fixed and immutable in time^2^,^3^. Indeed, it allows to capture a set of complex dynamics driving the evolution of links^5^,^6^,^7^,^8^,^9^,^10^,^11^ and to uncover the effects of such dynamics on processes unfolding on the networks’ fabrics^12^,^13^,^14^,^15^,^16^,^17^,^18^,^19^,^20^,^21^ (see ref. 3 for a recent review).

While social networks are shaped by a multitude of processes^22^, two particular mechanisms have been found to play central roles in their emergence and evolution^13^,^14^,^23^,^24^,^25^,^26^,^27^.

The first is the *strategy in activation of social ties*, i.e. the selection process driving the creation of a new connection, or the activation of an old one. Intuitively, social tie activation is not random. Empirical observations show that people tend to distribute a large fraction of their social acts towards already existing strong ties, while allocating a smaller amount of interactions to create new social relationships or to maintain weak ties^23^,^24^,^25^,^27^,^28^,^29^, *reinforcement process*. In other words, in time some connections are frequently used in repeated interactions, while others are not.

The second mechanism is *burstiness*, i.e. the activity of single individuals evolves through heterogeneous inter-event time distributions^30^,^31^,^32^,^33^,^34^,^35^,^36^,^37^,^38^. Furthermore, the propensity of individuals to be engaged in a social act per unit time is also heterogeneous. In fact, empirical measures on real datasets, capturing different types of social dynamics, show that activity is heterogeneously distributed among people^27^,^39^,^40^,^41^. In other words, not only individuals show heterogeneous propensities to be socially active, but their activation is bursty as well, and this bursty activity can dramatically influence the networks’ evolution.

Although the study of these mechanisms has been the focus of a range of works^13^,^14^,^23^,^24^,^25^,^26^,^27^, a general modeling framework is still missing. Such a framework would allow for the analytical characterization on how the interplay of heterogeneous activity patterns and tie selection mechanisms shapes the evolution of social networks, and in turn the processes taking place on their fabrics.

Here we introduce a model of time-varying networks that allows to simultaneously regulate the relative strength of bursty activity and tie activation strategy. We analytically solve the asymptotic behaviour of the model and find a non-trivial phase diagram ruling the interplay of the two processes. In particular, we observe a regime where burstiness governs the evolution of the network, and a different region where the the dynamics is completely determined by the process of ties selection. If the re-use of previously activated connections is sufficiently strong and people has a tendency to preferably contact the same social circle, burstiness sub-leads the reinforcement mechanism even in the presence of diverging inter-event time fluctuations, having no effect on the network evolution.

The theoretical results are validated showing that analytical predictions fit the empirical observations of two real world datasets: Physical Review B (PRB) collaboration network and Twitter mention network. Interestingly, PRB dataset belongs to a region of the parameter space where burstiness is suppressed and the statistics is Gaussian, while Twitter belongs to a regime of strong burstiness where non-Gaussian effects dominate the network evolution. Thus, the framework we propose can be used to classify the temporal features of real networks, and it could provide new insights on the effects of social mechanisms on spreading processes unfolding in social networks.

## Results

### Activity driven network with burstiness and tie activation strategy

In the framework of *activity driven networks*^24^,^27^,^39^, a network 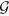 is formed by *N* nodes and each node *i* is assigned with an activity *a*_*i*_ drawn from an arbitrary distribution *F(a*_*i*_). The activity sets the activation rate of node *i*, i.e. the probability *a*_*i*_Δ*t* that a node active in time interval Δ*t*. In general, *F(a*_*i*_) is chosen to be a broad function reflecting the shape of the corresponding distribution in empirical observations^24^,^27^,^39^. Typically a power law distribution i.e. 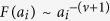 is observed for large activity.

The inter-event time *τ*_*i*_, i.e. the time between two subsequent activations of the agent *i*, is directly connected with the agent activity, since *a*_*i*_ = 1/〈*τ*_*i*_〉. With exception of ref. 31, which does not consider the ties selection process, the activity-driven models proposed so far considered a Poissonian distributed *τ*_*i*_. However, in social systems the inter-event time distribution of a single agent is strongly heterogeneous and usually spans over several orders of magnitude. In order to capture this bursty nature of human dynamics, we impose that the inter-event time *τ*_*i*_ for node *i* is drawn from a power-law distribution Ψ(*τ*_*i*_):


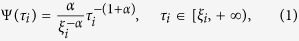


where the exponent *α* characterizes the distribution and *ξ*_*i*_ is a lower time cutoff. The latter represents the characteristic timescale for the node *i*, i.e. 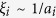, as the *γ*-th moment of the distribution Ψ(*τ*_*i*_) reads 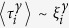. If also the *ξ*_*i*_ are heterogeneously distributed as


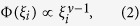


for small *ξ*_*i*_, we obtain a network in which the corresponding activity potential *a*_*i*_ is broadly distributed. In particular the activity distribution behaves as 
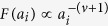
 for large *a*_*i*_. We note that, instead of introducing an agent dependent cut-off, different definitions are possible, e.g. considering a distribution of waiting times 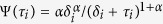, where 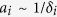 since 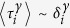. Our model, therefore, belongs to a novel class of activity driven networks, where the agent time scale is set by a parameter in the waiting time distribution.

When a node is active, it initiates interactions with other nodes in the network. This way the degree *k*_*i*_ of a node *i*, defined as the number of connected peers of *i* up to time *t*, is evolving in time. To model this evolution we build on the latest development of the model in which the selection of ties is driven by a reinforcement process^24^,^27^. In particular, if at time *t* a node *i* of degree *k*_*i*_ is active it will contact a new, randomly chosen node with probability *p*_*i*_(*k*_*i*_). Instead, with probability 1 − *p*_*i*_(*k*_*i*_) it reinforces a tie by contacting a node randomly chosen amongst the *k*_*i*_ already contacted agents. The form of *p*_*i*_(*k*_*i*_) has been measured and characterized^27^ in several datasets as:


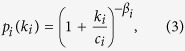


where *β*_*i*_ measures the strength of the reinforcement process, and *c*_*i*_ sets the characteristic number of ties that an individual is able to maintain before the reinforcement process takes place. Though simple, the reinforcement mechanism provides a reliable description of real world datasets and allows for analytical tractability.

In our simulations, initially, for each node *i* we set the integrated degree *k*_*i*_ = 0 and assign a lower cut-off *ξ*_*i*_ according to distribution Φ(*ξ*_*i*_) in Eq. (2). The evolution starts by extracting, for each node *i*, a time *τ*_*i*_ at which the node will get active for the first time. We then activate one node at a time accordingly to their next activation time. When active, an agent *i* selects a randomly chosen other agent in the network with probability *p(k*_*i*_) = (1 + *k*_*i*_/*c*)^−*β*^; in this case the value of *k*_*i*_ is increased by one both for the connecting and the connected nodes. Otherwise, with probability 1 − *p*_*i*_(*k*_*i*_), the agent *i* interacts with a randomly chosen neighbor node which has been already connected to *i*. For simplicity we fix *β* and *c* constant for all nodes. After each iteration the interaction of node *i* is removed and a new activation time is selected by an inter-event time *τ*_*i*_ drawn from the distribution in Eq. (1).

### Single agent approach

In the following we apply a single agent approximation, in which agents can only attach to other nodes and never get contacted. We can write the master equation (ME) for the degree evolution, and analytically solve it in the asymptotic limit of large times. The activity is fixed to the value 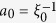 where *ξ*_0_ is the characteristic time of the considered agent. In particular, let us define *Q(k, t*) as the probability for an agent active at time *t* to have degree *k* right after *t*. The ME then reads as


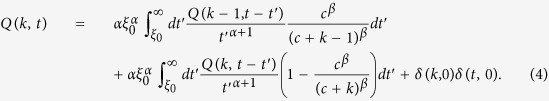


The first term accounts for the probability that the active agent contacts a new node at time *t*, while the second term represents the probability of connecting to an already contacted neighbor. We evaluate the probability *P(k, t*) for a node to have degree *k* at the time *t*, by integrating Eq. (4) over all the possible waiting time values, i.e.


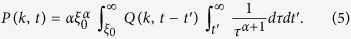


The solution to these equations for *P(k, t*) is, in the asymptotic regime (see Methods for details):


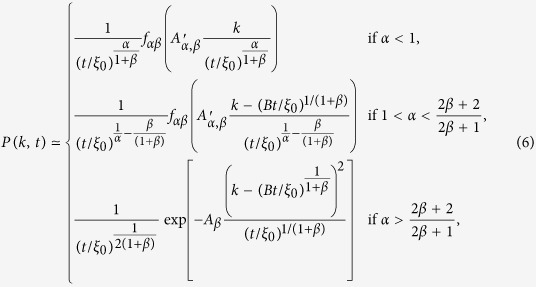


where *f*_*αβ*_(*x*) is a non-Gaussian scaling function (see ref. 42), and *A*′_*α,β*_, *A*_*β*_, and *B* are constants depending on *α* and *β*. Specifically, *A*′_*α,β*_ and *A*_*β*_ set the width of the non-Gaussian and Gaussian distributions respectively, while *B* is the drift velocity of the peak of *P(k, t*). In other words, the former constants modulate the heterogeneity of the interactions of an individual, while *B/ξ*_0_, sets the pace at which the degree of a node grows in time. How these constants can be calculated is described in the Methods section.

As a consequence of Eq. (6), the growth of the average degree 〈*k(t*)〉 is:


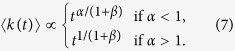


**Figure 1:**
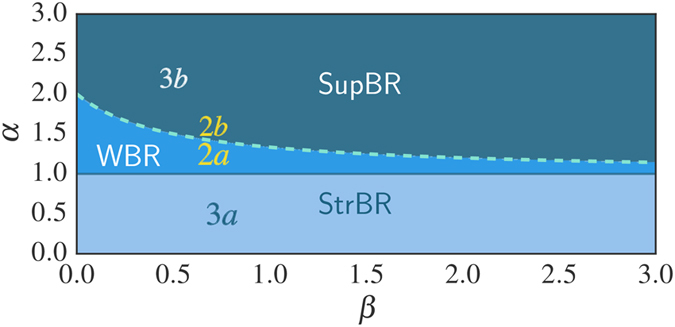
In the phase diagram we report the delimiting lines of the different scaling regions as found in Eq. (6). Evidencing the Strong Burstiness Regime (StrBR), the Weak Burstiness Regime (WBR), and the Suppressed Burstiness Regime (SupBR). We also show the parameters value of the simulations presented in Fig. 2 (yellow tags), and in Fig. 3 (white and blue tags).

**Figure 2:**
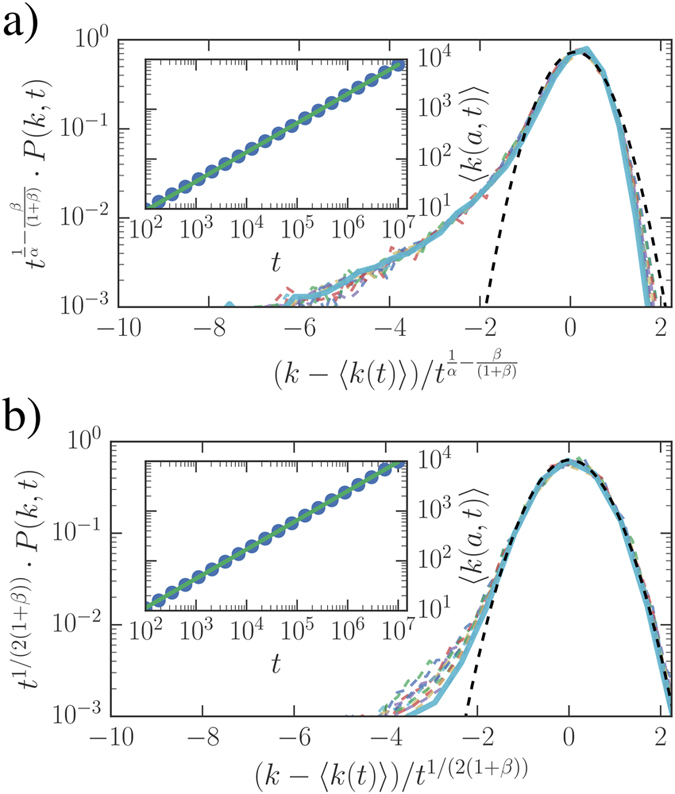
The scaling of the *P(k, t*) function for (**a**) *β* = 0.7, *α* = 1.35 (WBR region) and (**b**) *β* = 0.7, *α* = 1.6 (SupBR regime). In each plot we consider logarithmically spaced times between *t* = 10^4^ and *t* = 10^7^ averaged over 10^5^ representations of the single agent evolution. The curves referring to the longest time *t* = 10^7^ are shown in solid thick line, while shorter times are shown in dashed lines. A comparison with a normal distribution (black dashed lines) evidences a good agreement with the SupBR data (**b**) while it completely misses the WBR case (**a**). Insets plot the 〈*k(t*)〉 and the corresponding analytical prediction of Eq. (7) (green solid lines).

This solution leads to a dynamical phase diagram of the model, summarized in Fig. 1. For *α* < 1, in the **Str**ong **B**urstiness **R**egime (StrBR) burstiness strongly affects the dynamics. Here the scaling function *f*_*αβ*_(*x*) is not Gaussian and the exponent *α*/(1 + *β*), leading the growth of 〈*k(t*)〉, depends both on the burstiness and on the reinforcement exponents, *β* and *α* respectively. On the other hand, for *α* > (2*β* + 2)/(2*β* + 1), we have a **Sup**pressed **B**urstiness **R**egime (SupBR), where the dynamics is independent of *α*. In particular, the reinforcement driven behavior, described in ref. 27, is fully recovered with a Gaussian scaling function and a connectivity growing as *t*^1/(1+*β*)^. Finally, for 1 < *α* < (2*β* + 2)/(2*β* + 1) the average connectivity grows as *t*^1/(1+*β*)^ just as in the systems with suppressed burstiness. In this regime, the scaling function is not Gaussian and its scaling length depends on the burstiness exponent *α*. We call this phase the **W**eak **B**urstiness **R**egime (WBR). The non trivial dependence on *β* and *α* of the transition line between WBR and SupBR highlights the complex interplay between burstiness and tie reinforcement occurring for 1 < *α* < 2. Figure 2 shows that the curve *α* = (2*β* + 2)/(2*β* + 1) marks a transition from a Gaussian to a non Gaussian scaling function, providing a numerical support to the analytical asymptotic results. In particular, in Fig. 2(a) panel the left tail of the curve is slowly increasing with time, and the asymmetric distribution cannot be fitted with a Gaussian. On the other hand, in Fig. 2(b) we observe the opposite behavior: the long time curve is slowly converging to a normal PDF.

### Multi-agent simulations

**Figure 3:**
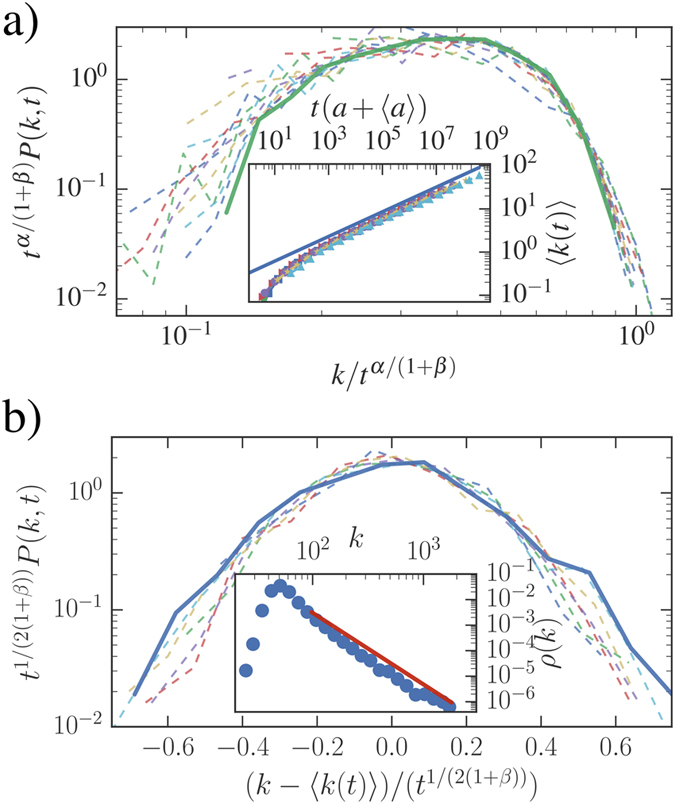
Numerical simulation of the full multi agent dynamics. (**a**) the rescaled *P(k, t*) distribution for *ξ*_*i*_ distributed with *ν* = 1.4, *α* = 0.5, *β* = 0.75. Curves refer to ten logarithmically spaced times between *t* = 10^5^ and *t* = 10^8^ (dashed lines, the longest time is shown in solid line). The data correspond to the StrBR regime. In the inset we show the growth of average degree for different activity classes (symbols) rescaled as *t* → *t(a* + 〈*a*〉). The analytical prediction of Eq. (7) is shown for comparison (blue solid line). (**b**) the *P(k, t*) distribution for *ν* = 1.2, *β* = 0.5 and *α* = 2.2, at seven logarithmically spaced times between 10^4^ ≤ *t* ≤ 10^6^. Inset compares the degree distribution at the final simulation time (circles) with the analytical prediction (solid line) of Eq.(8).

Interestingly, the single-agent model provides a qualitatively correct description even of the multi-agent case, where different agents display different activities (see Fig. 3). In particular, one can suppose that the degree distribution Eq. (6) holds for each node of the system if one replaces *a*_0_*t* with (*a* + 〈*a*〉)*t*. In this case *at* and 〈*a*〉*t* represent the contribution to the growth of the degree induced by the node activity and by the activity of the rest of the network respectively (see refs 27,43 for an explicit calculation in the case without burstiness). Simulations confirm that the approximation works quite well, however, larger evolution times are required for observing the asymptotic scaling behavior. The same hypothesis allows to evaluate the degree distribution among different agents. In particular, if the activity *a* is distributed according to Eq. (2), at a given time *t* the degrees, for large *k*, are distributed as:


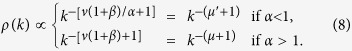


As shown in Fig. 3(b) inset, the simulation results are well described by the asymptotic behavior in Eq. (8).

### Real world dataset

We now compare the asymptotic behavior predicted by the model with real world datasets. In particular we consider Twitter Mentions Network and the citation network of PRB, see Methods for details.

**Figure 4:**
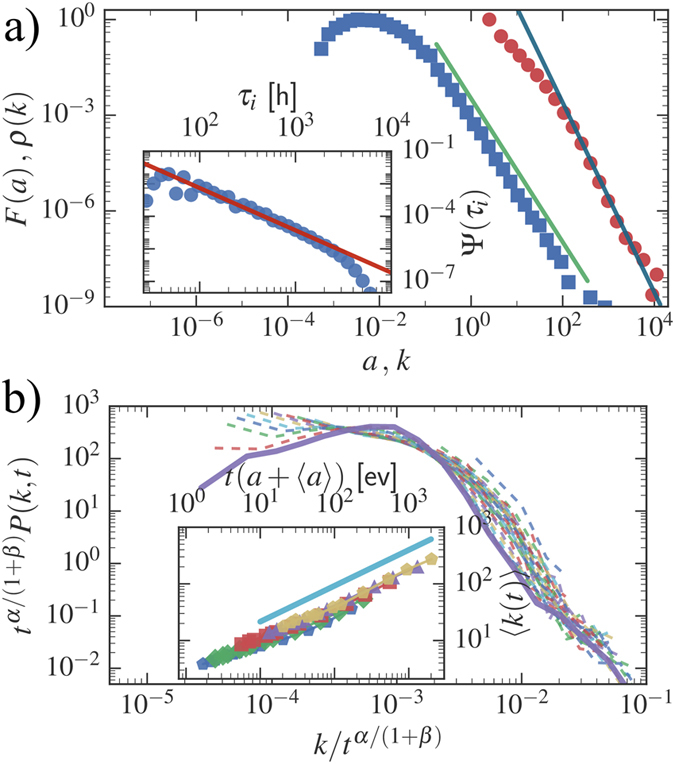
Twitter mentions dataset. (**a**) The activity distribution *F(a*) (blue squares) fitted as *F(a*) ∝ *a*^−(1+*ν*)^ (green solid line) with *ν* = 1.24(3) and the degree distribution *ρ(k*) (red circles) with the predicted behavior *ρ(k*) ∝ *k*^−(1+*μ*′)^ (blue solid line) of Eq. (8) giving *μ*′ = 1.94, in good agreement with the measured value 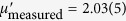. In the inset we plot the waiting-time distribution Ψ(*τ*_*i*_) (blue circles) and the fit 
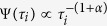
 (red solid line) giving *α* = 0.946(14). (**b**) The distribution *P(a, k, t*) for a selected activity class, the degree *k* is rescaled dividing by *t*^*α*/(1+*β*)^ where *β* = 0.47 has been found in ref. 27 and *α* was evaluated in the upper panel. Inset shows the average degree growth 〈*k(t*)〉 for different activity classes *a* (symbols) rescaling time as *t* → *t(a* + 〈*a*〉). The predicted behavior of Eq. (7) is shown for comparison (green solid line).

**Figure 5:**
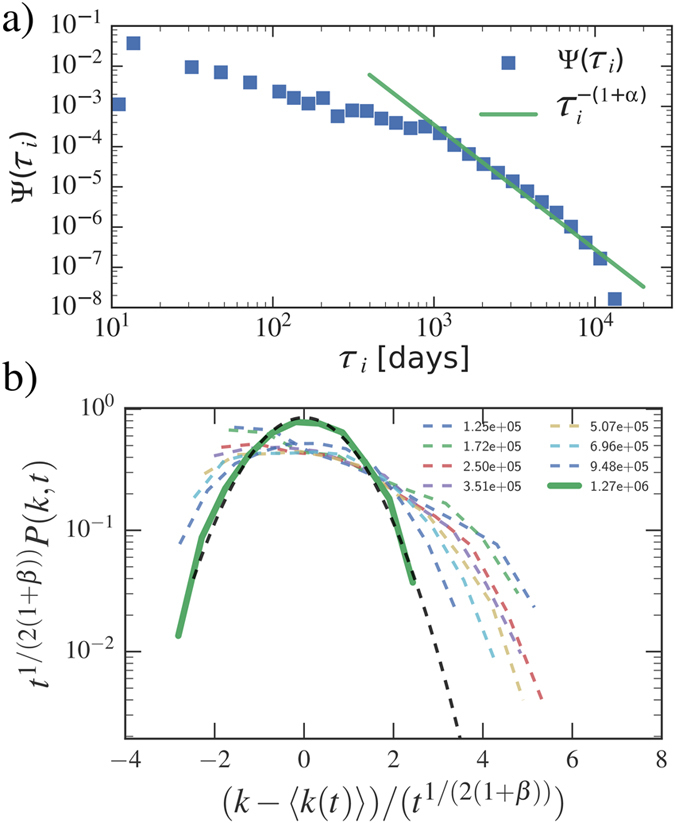
(**a**) The waiting-time distribution Ψ(*τ*_*i*_) for the co-authorship network of the PRB journal (squares). We also show the fitting curve of the right tail 
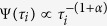
 giving *α* = 2.06(10). Given these results the system falls above the *α* = (2*β* + 2)/(2*β* + 1) curve, thus falling in the SupBR regime. In (**b**) we show the rescaled *P(k, t*) distribution at different evolution times (coloured dashed lines) with the longest time distribution in green solid line. The normal distribution fitting the empirical data is shown for comparison (black dashed line).

We measure the *α* exponent leading the inter-event time distribution with the procedure found in ref. 44. In Twitter the distribution approximately follows a power-law (see Fig. 4(a) inset) with 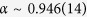, while the exponents 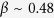 and 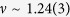 characterizing the tie activation strategy and the activity distribution have been measured in ref. 27. Since for Twitter *α* < 1, we expect the system to fall in the StrBR region. In Fig. 4(b) we verified that, at different evolution times, *P(k, t*) is not Gaussian. Moreover, the dynamical scaling of *P(k, t*) and the asymptotic behaviors of 〈*k(t*)〉 and *ρ(k*) follow the predicted behavior in Eqs (7,8). In the PRB dataset we find *α* = 2.06(10) as shown in Fig. 5(a). This system, therefore, falls in the SupBR region of the phase diagram. Consistently, in Fig. 5(b) we show that the analytical Gaussian prediction of Eq. (6) correctly catches the asymptotic behavior of *P(k, t*). In this case the behavior of 〈*k(t*)〉 and *ρ(k*) is described by the model without burstyness as shown in ref. 27.

## Discussion

In summary, we introduced a new model, which is able to capture two key aspects driving the evolution of social networks: tie activation strategies and burstiness. We solved the ME of the model in the large time regime and in unsaturated degree approximation 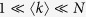, analytically exploring a complex phase space, where changes in the relative importance between the two mechanisms are linked to different degree distributions and emerging dynamics.

The proposed framework allows to classify the dynamical features of real social networks and thus anticipate their effects on spreading processes taking place on their fabrics. In particular, the model could help to clarify the role of burstiness on contagion phenomena, which is currently subject of a heated debate. It can also potentially be extended further by including other social processes such as the presence of communities or aging of nodes. Furthermore, in real world networks links appear with finite lifetimes and nodes typically enter or leave from the system during the network evolution. Our modelling framework does not include these dynamics. Earlier results^45^ of node removal processes in activity-driven network models suggest that after an initial period the network arrives to a stationary state, where its overall characteristics become time invariant. We leave the inclusion of such important processes in our model for future investigations.

## Methods

### Dataset

The analyzed dataset is the Twitter fire-hose (i.e. all the citations done from all the users) from January the 1st to September the 31st of 2008. The dataset consists of 536,210 nodes performing about 160 M events and connected by 2.6 M edges.

Since the data are daily aggregated we infer the inter-event time distribution for 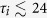 h by assuming the events done by a node within a single day to be homogeneously distributed during the 24 hours of the day. As we are measuring the *α* exponent leading the 
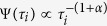
 in the right tail of the distribution this assumption does not change the resulting *α*. The *α,ν*, and *μ* exponents are measured using the procedure found in ref. 44 and reads *α* = 1.946(14), *ν* = 1.24(3), and *μ*′ = 2.03(5) respectively.

The PRB dataset contains data from from January 1970 to December 2007. The data are cleaned so as to not take into account the papers with a single author. Moreover, we do not include large collaborations in our analysis (papers with more than ten authors). Here data are naturally aggregated by month and we remark that to avoid problems of name disambiguation we used the data generated from ref. 46. To obtain the exponent *α* we apply to Ψ(*τ*_*i*_) the same procedure^44^ of the Twitter case. In PRB we measure *α* = 2.06(10) so that the dataset belongs to the suppressed burstiness regime.

In both cases, to measure the reinforcement process and specifically the *β* exponent we measure the attachment probability on nodes featuring similar stories, i.e. with a comparable activity *a*_*i*_ (i.e. the number of events actually engaged by the node *i*) and final degree *k*_*i*_ (see ref. 27 for details). We then refer the reader to the work in ref. 27 for the *F(a*) and the *ρ(k*) distributions analysis.

### Master Equation in the single agent approximation

In the calculation of *P(k, t*) within the single agent approximation we perform the Fourier Transform of Eq. (4):


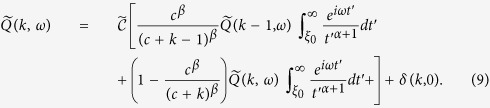


where 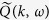 is the transform of *Q(k, t*) and 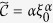. By taking the limit *k* → ∞ of Eq. (9) we end up with


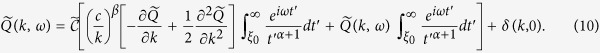


The issue is now to compute the integral appearing in Eq. (10). For *ω* → 0 we have


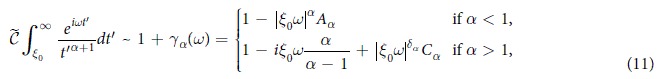


where 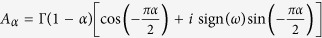 and


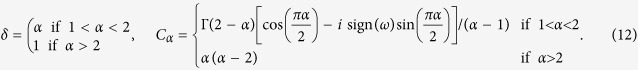


In Eq.(10) keeping the leading orders for large *k* and small *ω* the first integral can be approximated to the zero-th order in *ω* while the second should be expanded as Eq. (11):


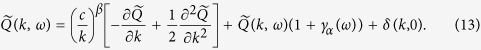


We now introduce the new variable *h* = *k*^1+*β*^, so that 
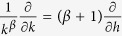
 and for large *h* 
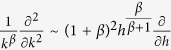
. We then have:


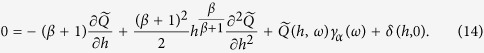


Introducing now 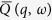, the Fourier Transform of 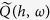 with respect *h*, we have:


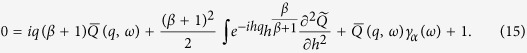


Let us now perform the Fourier Transform with respect to *h* and *t* also for Eq. (5). We get:


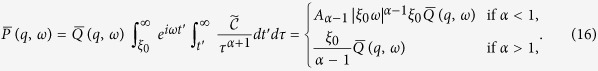


For *α* < 1, we can plug Eq. (16) into Eq. (15) and keeping the first order for *ω* → 0 and *q* → 0 we have:


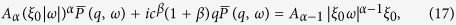


so that:


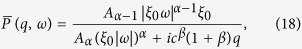


This equation is the same of Eqq. (8) discussed in details in ref. 42, so that we can extract the asymptotic solution:


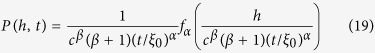


where *f*_*α*_ is a Lévy function. Reintroducing the degree variable *k* = *h*^1/(1+*β*)^ we obtain the first of Eq. (6).

For *α* > 1, we plug again Eq. (16) into Eq. (15) and we have:


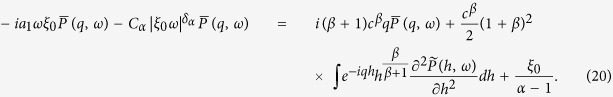


where *a*_1_ = *α*/(*α* − 1). Here we have to take into account the second order for small *ω* and *q*. Indeed the first order term in *q* can be subtracted by introducing the variable 
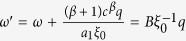
 (where *B* is a numerical constant). In the direct space, this corresponds to a shift of the *h* variable *h*′ = *h* − *vt* with 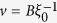. Introducing in (20) the shifted variables, we now get:


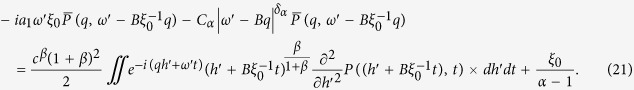


Eq.(21) displays different behaviors whether, for *q* → 0 and *ω* → 0, the term 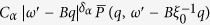 is dominant with respect to the integral. This can be discussed introducing a scaling hypothesis in Eq.(21). In particular, we expect 
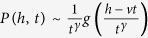
 with *γ* < 1. In the Fourier space we get:


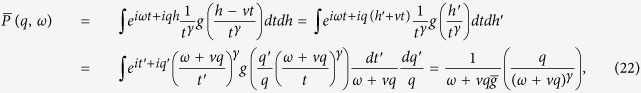


Comparing the second integral with the final result we obtain the scaling form of the Fourier transform of *P(h*′ + *vt, t*):


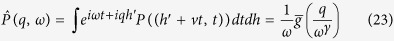


Let us now focus on the integral in Eq.(21). First we can approximate 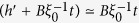, as we expect 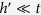. Then we can integrate it by parts and write 
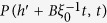
 as 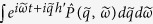 getting:


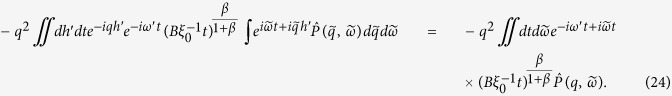


Now we insert the scaling form of 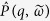 of Eq. (22). Introducing first *ω*′*t* = *z* and then 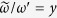, we get:


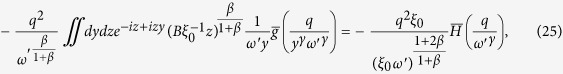


where 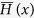 is a new scaling function. Putting Eq.s (25) and (22) (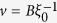) in Eq. (21) we get:


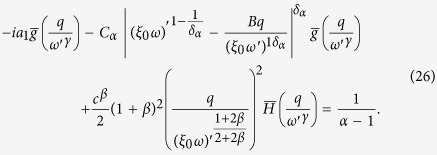


Clearly from Eq.(26) we have 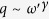 and, since 
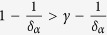
, we get that 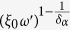 is always sub-leading with respect 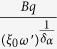. From Eq. (26) we get that *γ* can have the following values: 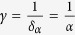 if the term 
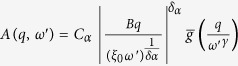
 dominates over 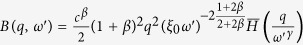; or 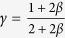 if *B(q,ω*′) dominates.

In particular, if 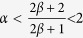, we have 
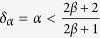
 and 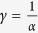. In this case, indeed, since 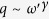, we get 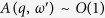, while 
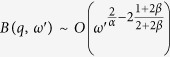
. The scaling form of *P(k, t*) can be recovered taking into account that the maximum of *P(h, t*) grows as *h* = *vt* and we can expand *P(h, t*) with respect to the small variable 
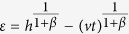
:


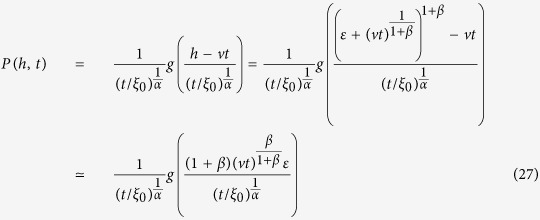


where the dependence on *ξ*_0_ is determined by the fact that in Eq. (26) *ω*′ always occurs through *ω*′*ξ*_0_. Let us change the variable *h* into 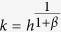 taking into account that 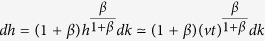. We get


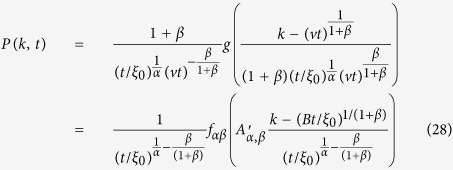


So we obtain the second of Eq. (6).

Otherwise, if 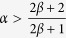, 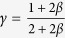, 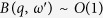 dominates over 
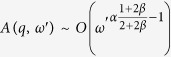
. In particular in Eq. (20) we can neglect the term 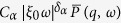. So that returning to the direct space and reintroducing the variable *k* we get:


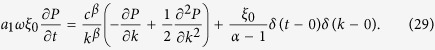


Eq. (29) has been studied in ref. 27 showing that *P(k, t*) is a Gaussian function described by the third of Eq. (6).

### Degree distribution *ρ(k*)

The degree distribution *ρ(k*) can be evaluated in the hypothesis that *P(k, t*) display the behavior of Eq. (6) even in the multi-site case. Clearly, at fixed time *t*, we have:


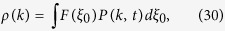


where *F(ξ*_0_) is the distribution of the lower-cut off *ξ*_0_ of the inter-event times. We will consider a distribution of *ξ*_0_ going as *F(ξ*) ∝ *ξ*^*ν*−1^ corresponding to an activity distribution behaving as: *F(a*) ∝ *a*^−(*ν*+1)^ indeed *a* ∝ *ξ*^−1^. For the case *α* < 1 we get:


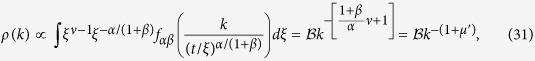


where 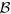 is a constant. For *α* > 1 we note that in the large *t* limit the degree distributions tends to 
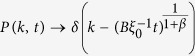
 so that we obtain:


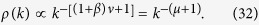


## Additional Information

**How to cite this article:** Ubaldi, E. *et al*. Burstiness and tie activation strategies in time-varying social networks. *Sci. Rep.* **7**, 46225; doi: 10.1038/srep46225 (2017).

**Publisher's note:** Springer Nature remains neutral with regard to jurisdictional claims in published maps and institutional affiliations.
